# Characteristics of criminal cases against physicians charged with opioid-related offenses reported in the US news media, 1995–2019

**DOI:** 10.1186/s40621-020-00277-8

**Published:** 2020-10-01

**Authors:** Julia B. Berman, Guohua Li

**Affiliations:** 1 Department of Anesthesiology, Columbia University Vagelos College of Physicians and Surgeons, 622 West 168th Street, PH5-534, New York, NY 10032 USA; 2 Department of Epidemiology, Columbia University Mailman School of Public Health, 622 West 168th Street, PH5-534, New York, NY 10032 USA

**Keywords:** Drug overdose, Drug trafficking, Opioid epidemic, Medical fraud, Prescription opioids

## Abstract

**Background:**

Pharmaceutical companies and drug distributors are intensely scrutinized in numerous lawsuits for their role in instigating the opioid epidemic. Many individual physicians have also been held accountable for activities related to prescribing opioid medications. The purpose of this study was to examine the epidemiologic patterns of criminal cases against physicians charged with opioid-related offenses reported in the US news media.

**Methods:**

We searched the Nexis Uni® database for news media reports on physicians who had been arrested, indicted or criminally charged for illegally prescribing opioids between January 1995 and December 2019. Data collected from the news media reports include defendant’s age, sex, clinical specialty, type of crime and legal consequences.

**Results:**

The annual number of criminal cases against physicians charged with opioid-related offenses reported in the US news media increased from 0 in 1995 to 42 in 2019. Of the 372 physician defendants in these criminal cases, 90.1% were male, 27.4% were 65 years and older, and 23.4% were charged in Florida. Of the 358 physician defendants with known clinical specialty, 245 (68.4%) practiced in internal medicine, family medicine, or pain management. Drug trafficking was the most commonly convicted crime (accounting for 54.2% of all convicted cases), followed by fraud (19.1%), money laundering (11.0%) and manslaughter (5.6%). Of the 244 convicted physicians with known sentences, 85.0% were sentenced to prison with an average prison term of 127.3 ± 120.3 months.

**Conclusions:**

The US news media has reported on an increasing number of opioid-related criminal cases against physicians from a wide variety of clinical specialties. The most commonly convicted crime in these cases is drug trafficking, followed by fraud, money laundering, and manslaughter.

## Background

Misuse and overdose of opioids is a public health crisis in the United States and many other countries. From 1999 to 2018, nearly 450,000 people in the United States died from overdoses involving prescription opioids (Centers for Disease Control and Prevention (CDC), 2020; Hedegaard et al., 2020). The opioid epidemic was triggered in the 1990s by physician overprescribing of opioid analgesics (Brady et al., 2014; Chihuri and Li, 2019; Hedegaard et al., 2020; Li and Chihuri, 2020). While a significant proportion of those prescriptions might be well-intentioned, clinical treatment for a variety of pain syndromes, there are some medical professionals who prescribe and dispense opioid prescriptions for personal profit. It is illegal for physicians to prescribe a controlled substance with no legitimate medical purpose and outside the usual course of professional practice (Rigg et al., 2010). Such physicians can be charged with drug trafficking and face severe legal consequences (Rigg et al., 2010). Physicians and clinics responsible for these illegal prescriptions are colloquially known as “pill mills,” common characteristics of which include physicians prescribing narcotics without conducting physical examinations or consulting medical records, allowing patients to pick their own medicine, treating pain with pills only, prescribing a set number of pills and giving the patient a specific date to return for more, accepting cash only and crowded waiting rooms (Rigg et al., 2010). These physicians have played an important part in perpetuating the opioid epidemic in the United States (Kennedy-Hendricks et al., 2016).

Reducing opioid prescriptions, particularly illegal opioid prescriptions, could help limit the overall quantity of opiates being distributed to the communities and thereby decrease the availability and consumption of these addictive drugs. In recent years, pharmaceutical companies and drug distributors have been intensely scrutinized in numerous lawsuits for their role in instigating the opioid epidemic. Many individual physicians have also been held accountable for activities related to prescribing opioid medications. The purpose of this study was to examine the epidemiologic patterns of criminal cases against physicians charged with opioid-related offenses reported in the US news media.

## Methods

Nexis Uni® is an electronic database that houses an archive of public record documents, such as full-text newspapers, business and legal publications, and journals. An initial search was conducted on the database with search terms “overprescribing opioids”, “overprescribe opioids”, “overprescribed opioids”, and “pill mill.” The search was refined with terms (“doctor” or “dr.” or “MD”) AND (“sentenced” or “charged” or “convicted” or “sentence” or “charge”) AND (“years” or “fined” or “months” or “prison”) AND (“pill mill”) AND (“opioid” or “narcotic” or “drugs”) and limited to publications released after January 1st, 1995. The database was queried with these terms so as to identify news media articles that reported incidents of physicians overprescribing opioids since 1995. The search yielded over 2000 results of full-text newspapers and court reports, which were manually examined to extract a list of physicians, who, according to the news media reports, had been arrested, criminally charged, or indicted for illegally prescribing opioids. Duplicate physicians were manually excluded by reviewing names.

Next, individual searches for each physician were executed on Nexis Uni®; the database was queried using only the physician name, in order to produce narrower, more focused results. From these searches, the following information was ascertained: name, state in which the incident occurred, type of medical facility in which the physician worked (hospital, private practice, etc.), medical specialty, age at the time of incident, sex, type of criminal charge, and outcome of the legal proceeding. Multiple news media reports were examined for each physician. Further, these articles uncovered other physicians who fit the search criteria but had not come up in the initial search; these physicians were added to the list. Searches on Google News were carried out for physicians with incomplete information. Data collected from news media reports for each physician involved in these incidents were analyzed using descriptive statistics such as frequencies, percentages, means, and standard deviations.

## Results

**Fig. 1:**
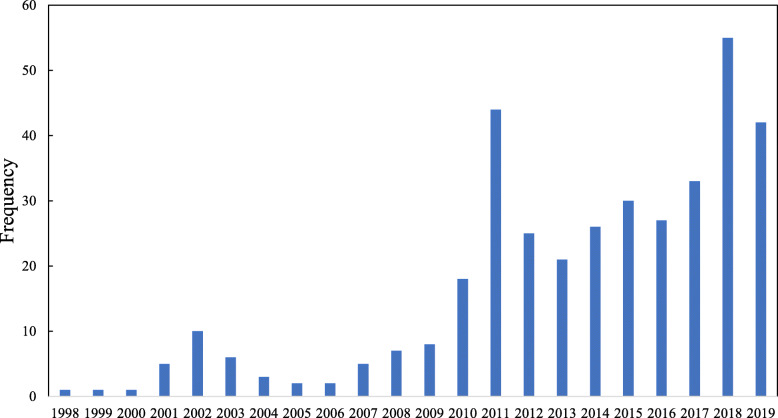
Annual Frequency of Criminal Cases against Physicians Charged with Opioid-Related Offenses Reported in the US News Media, 1995–2019

**Fig. 2:**
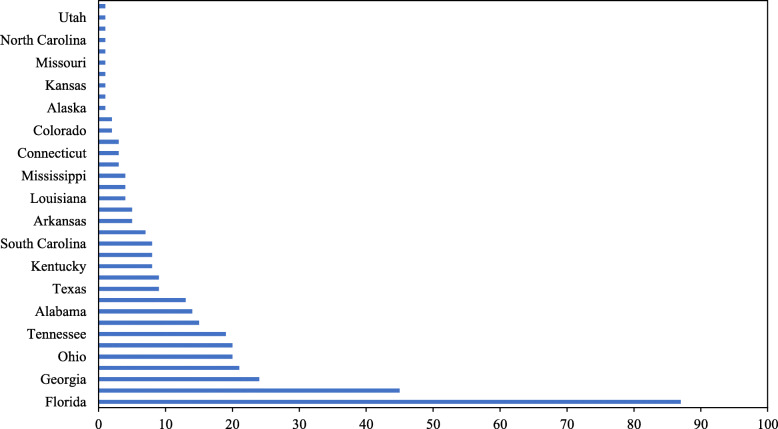
Frequency of Criminal Cases against Physicians Charged with Opioid-Related Offenses Reported in the US News Media by State, 1995–2019

During January 1995 through December 2019, the US news media reported on a total of 372 physicians who were involved in opioid-related criminal cases, exclusive of reports on 12 physicians involved in civil lawsuits for negligent opioid prescribing behaviors. There were no opioid-related criminal cases against physicians reported in the US news media between 1995 and 1998. Of the 372 criminal cases covered by the US news media, 231 (86.3%) occurred between January, 2010 and December, 2019 (Fig. 1), and nearly a quarter (23.4%) occurred in Florida, followed by Pennsylvania (12.1%), Georgia (6.5%), West Virginia (5.6%), Ohio (5.4%), New York (5.4%) and Tennessee (5.1%) (Fig. 2).

**Fig. 3:**
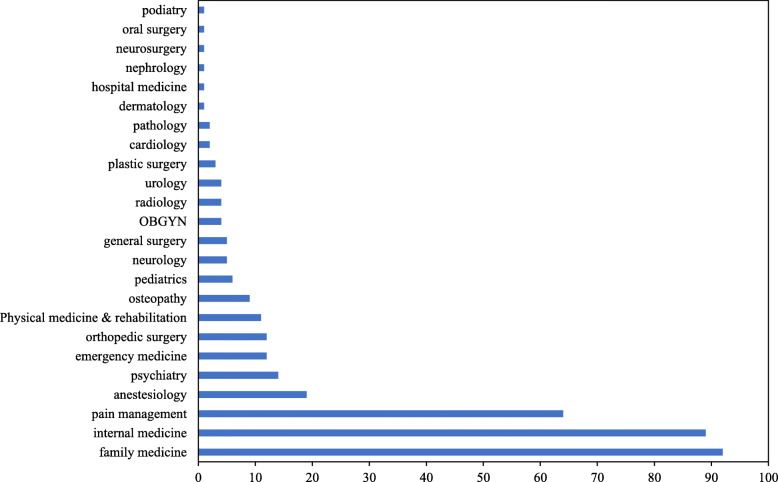
Frequency of Criminal Cases against Physicians Charged with Opioid-Related Offenses Reported in News Media by Clinical Specialty, United States, 1995–2020

The vast majority (90.1%) of physicians involved in the criminal cases were male. Ages of the physicians ranged from 33 to 87 (mean = 58.6 ± 10.7 years), with 27.4% being 65 years and older. Information on clinical specialty was available for 358 physicians. These physicians practiced in a variety of clinical specialties, with 25.7% in family medicine, 24.9% in internal medicine, and 17.9% in pain management (Fig. 3).

Nearly all the physicians prosecuted for opioid-related offenses (98.8%) worked in private practices. Of the 250 cases with known outcomes, representing 67.2% of the 372 cases, 246 physicians (98.4%) were convicted and 4 (1.6%) were acquitted. Drug trafficking accounted for 54.2% of the crimes convicted, followed by fraud (19.1%), money laundering (11.0%), manslaughter (5.6%), and other or unknown (10.1%). Of the 244 convicted physicians with known sentences, 85.0% served time in prison, with an average prison term of 127.3 (± 120.3) months and an average probation term of 65.5 (± 61.0) months.

## Discussion

Results of this study indicate that criminal cases against physicians charged with opioid-related offenses increased over time between 1995 and 2019, with the majority of the cases occurring between 2010 and 2019. This temporal pattern is generally consistent with the time trend of the opioid epidemic in the United States (Skolnick, 2018; Hedegaard et al., 2020). The geographic pattern of the criminal cases against physicians, however, might be more reflective of enforcement intensity on the state level. For instance, Florida passed laws in 2010 and 2011 that considerably reduced physicians’ ability to distribute opioids at the site of care, and subsequently, Florida law enforcement implemented initiatives to arrest and prosecute physicians who did not abide by these laws, resulting in an immediate hike in criminal cases against physicians charged with opioid-related offenses (Kennedy-Hendricks et al., 2016).

Our study also found that the vast majority of physicians involved in these criminal cases were male and worked in private practice. While men are known to be more prone than women to commit crimes (Burton et al., 1998), private practice presents a work environment that is less strictly regulated and supervised than hospitals. It is also noteworthy that over a quarter of the criminal cases included in the study involved physicians aged 65 years and older. As of 2018, about 17% of US physicians were older than 65 years (Elflein, 2019). The overrepresentation of older physicians in opioid-related criminal cases is likely multifactorial, including lower awareness of trends in pain management and heightened risk associated with the private practice environment. Further, physicians involved in opioid-related criminal cases reported in the US news media came from a broad range of clinical specialties, underscoring the needs for continuing attention to aberrant opioid prescribing patterns in all specialty areas.

This study is limited by the availability of information in the news media reports. Although news media has long been used for sentinel surveillance on infrequent, newsworthy events, such as drowning involving children with autism (Guan and Li, 2017) and alcohol-impaired airline pilots (Kraus and Li, 2006), they tend to capture the more severe incidents (Rainey and Runyan, 1992). Therefore, our findings are likely biased toward criminal cases involving serious offenses. Finally, our study was limited to physicians criminally charged with opioid-related offenses. Physicians involved in civil cases related to opioid prescribing practice were not included in this study. It is necessary to point out that many advanced healthcare practitioners other than physicians are also prescribers of opioid analgesics and may have been criminally charged during the study period as well.

## Conclusions

Our study sheds light on the issue of legal accountability of physicians amid the opioid epidemic. The results suggest that there have been increasing prosecutions for opioid-related crimes against physicians in the past decade, with severe legal consequences such as long-term prison sentences. The most commonly convicted offense in these criminal cases is drug trafficking (i.e., illegal distribution of controlled substances), followed by fraud, money laundering, and manslaughter.

## Data Availability

Data analyzed in the current study were abstracted from news media reports and are available from the corresponding author upon request.
